# The complete chloroplast genome sequence of *Asplenium komarovii* Akasawa, a rare fern in South Korea

**DOI:** 10.1080/23802359.2021.1961624

**Published:** 2021-09-22

**Authors:** Namjoo Heo, Seona Yun, Danilo D. Fernando

**Affiliations:** Department of Environmental Biology, State University of New York College of Environmental Science and Forestry, Syracuse, NY, USA

**Keywords:** Rare species, chloroplast genome, *Asplenium scolopendrium*, *Asplenium komarovii*

## Abstract

We sequenced the complete chloroplast genome of *Asplenium komarovii* Akasawa (syn: *Asplenium scolopendrium* L. subsp. *japonicum* (Komarov) Rasbach, Reichstein & Viane), which is designated as a rare species in South Korea. The complete chloroplast genome is 149,393 bp in total length and comprised of the following regions: large single copy (82,464 bp), small single copy (21,345 bp), and a pair of inverted repeats (22,792 bp). The overall GC content is 40.9% and the genome encoded a total of 115 genes, including 84 protein-coding, 27 transfer RNA, and 4 ribosomal RNA genes. Phylogenetic analysis based on 21 representative chloroplast genomes of the suborder Aspleniineae (and one outgroup) indicates that Aspleniaceae is monophyletic and sister to Diplaziopsidaceae, with Rhadidosoraceae as the basal group in this three family clade. *Asplenium komarovii* is sister to *A. nidus* and *A. prolongatum* with strong bootstrap support. The chloroplast genome of *A. komarovii* will be useful in establishing its relationships within the *A. scolopendrium* complex, which is currently unresolved.

*Asplenium komarovii* Akasawa (synonym: *Asplenium scolopendrium* L. subsp. *japonicum* (Komarov) Rasbach, Reichstein & Viane) is distributed in Korea, Japan, China, and Russia. In Korea, it is found in the inland regions of Gangwon-do and Jeolla-do, and the two islands of Ulleung and Jeju (Ok and Yoo 2012), but most populations are very small and fragmentated. Therefore, *A. komarovii* has been protected as a rare plant by the Korea Forest Research Institute since 1997.

*Asplenium scolopendrium* is a temperate evergreen fern species, which occurs in Europe, North America, and East Asia. The taxonomic history of this species is extensive and contentious, and currently with 5 major classifications: two European (*A. scolopendrium* var. *scolopendrium* and *A. scolopendrium* subsp*. antri-jovis*), two American (*A. scolopendrium* var. *americanum* and *A. scolopendrium* var*. lindenii*), and one Asian (*A. scolopendrium* subsp. *japonicum*). The complexity of their variations with respect to morphology, ploidy levels (2x in var. *scolopendrium*; 4x in var. *americanum* and subsp. *japonicum*), ecological preferences, and geographic distribution has created considerable scientific interest as well as taxonomic ambiguity (Emmott 1964; Futyma 1980; Cinquemani et al. 1988). Even if morphological characters show differences among varieties or subspecies, they are usually not decisive. This indicates that successful taxonomic treatment of the *A. scolopendrium* complex requires multiple criteria. Apparently, even with the current used of both morphology and ploidy level screening, the ambiguity in identification and classification remains. Thus, the incorporation of additional traits is necessary and use of DNA markers will provide decisive identification and classification, and such can be achieved with the complete chloroplast genome sequence of members of the *A. scolopendrium* complex. This report on the chloroplast genome of *A. komarovii* is going to pave the way for the establishment of its phylogenetic relationships with other *Asplenium* species, particularly the members of the *A. scolopendrium* complex. Information on the chloroplast genome of *A. komarovii* is also necessary to provide new insights for future studies such as marker development for inter- and intra-species identification, population genetics, and reconstruction of evolutionary history.

Here, we report the complete chloroplast genome of *A. komarovii*. We obtained a leaf sample from the Korea National Arboretum (voucher specimen KHB1469365, N33°24′1″ E126°24′39″). DNA was isolated using the DNeasy Plant mini kit (Qiagen, Germany) and sequenced with the Illumina platform (Macrogen, Korea). A total of 71,538,770 reads were obtained and assembled *de novo* with Velvet v. 1.2.10 using multiple k-mers (Zerbino and Birney 2008). The genes for annotation were identified using DOGMA (Wyman et al. 2004 ) and tRNAscan-SE software with default settings (Schattner et al. 2005). The start and stop codons were manually corrected and further verified by homology searches using BLAST (Altschul et al. 1990). The complete chloroplast genome of *A. komarovii* has been deposited in GenBank under the accession number MZ064529. For phylogenetic analysis, the chloroplast genome of *A. komarovii* and 21 other species belonging to the Aspleniineae were used with *Hypodematium crenatum* from Polypodiineae as the outgroup. Maximum-likelihood analysis was conducted using IQ-TREE v.1.6.7 (Nguyen et al. 2015).

The chloroplast genome of *A. komarovii* is 149,393 bp in size. It exhibits a circular quadripartite structure comprised of large single copy (LSC) and small single copy (SSC) regions, which are 82,464 and 21,345 bp long, respectively. They are separated by a pair of inverted repeats (IRA and IRB) which are each 22,792 bp long. The GC contents of the LSC, SSC, and IR regions are 39.7, 37.7, and 44.7%, respectively. *Asplenium komarovii* has a total of 115 unique genes which are subdivided into 84 protein-coding, 27 tRNA and four rRNA genes.

The phylogenetic tree shows that Aspleniaceae is monophyletic and sister to Diplaziopsidaceae, with Rhachidosoraceae as the basal group among these three families that form as a clade (Figure 1), which is supported by strong bootstrap values (BS = 100). This clade is distinct from the rest of the Aspleniineae, which also have mostly strong bootstrap supports (BS = 100). These results are generally consistent with the previous studies (Cui et al. 2019; Lehtonen and Cárdenas 2019). *Asplenium* species shared a recent common ancestor (BS =100) and that *A. komarovii* is closely related to *A. nidus* and *A. prolongatum* (Figure 1). The relationship of *A. komarovii* with members of the *A. scolopendrium* complex will be examined with the availability of the complete chloroplast genome sequence from *A. scolopendrium* var s*colopendrium* and var. *americanum* (Heo, Yun and Fernando, unpublished data).

**Figure 1.**
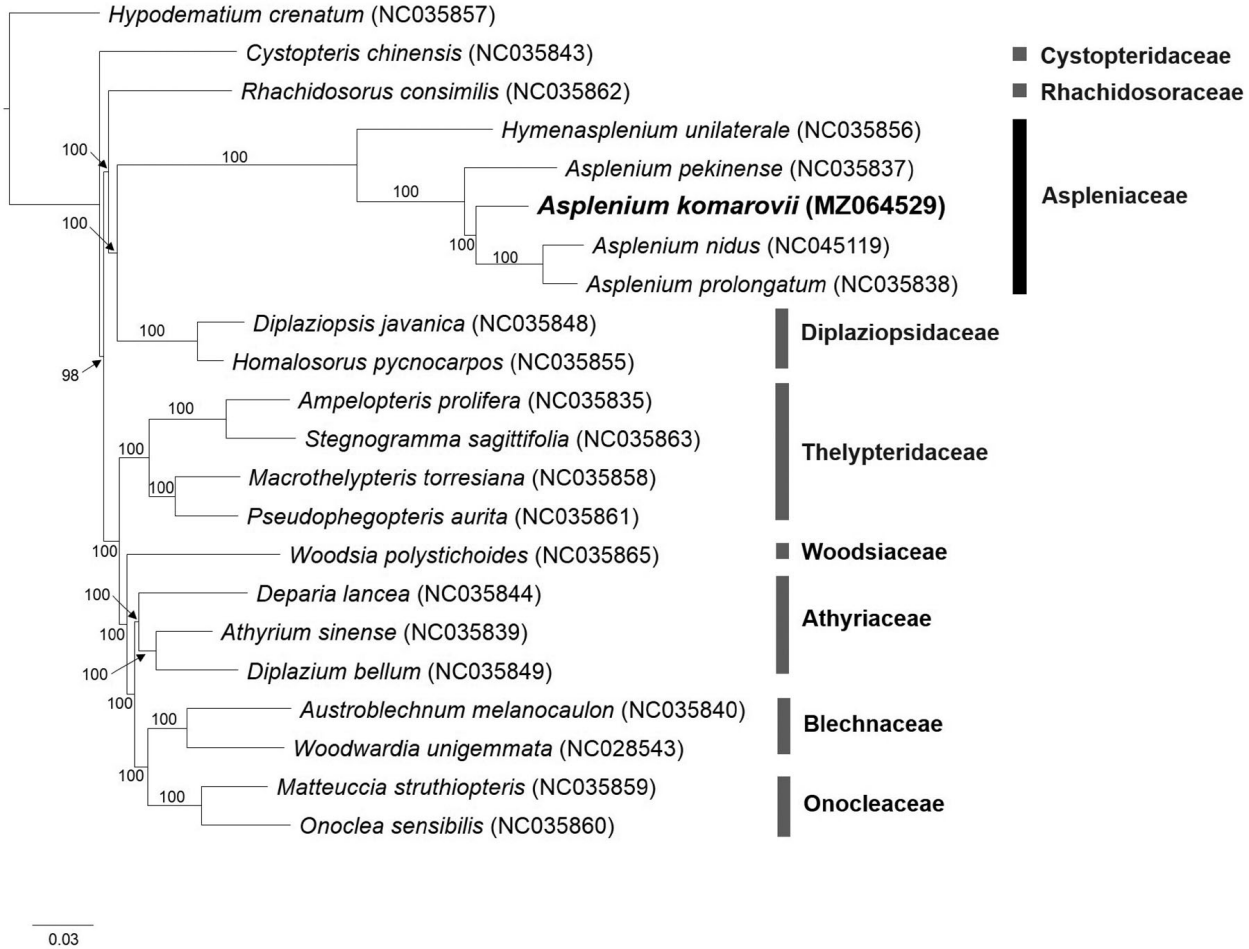
Phylogenetic tree of Aspleniineae based on maximum likelihood. Bootstrap support values are based on 1000 replicates and shown on each node.

## Data Availability

The genome sequence data that support the findings of this study are openly available in GenBank of NCBI at https://www.ncbi.nlm.nih.gov/ under the accession no. MZ064529. The associated BioProject, SRA, and Bio-Sample numbers are PRJNA726739, SRR14381415, and SAMN18957913, respectively.
